# Saunders’ Medical Hand-Atlases

**Published:** 1902-04

**Authors:** 


SAUNDERS' MEDICAL HAND-ATLASES.—Atlas and epitome of bacteriology; a text-book of special bacteriologic diagnosis. By Professor Dr. K. B. Lehmann, Director of the Hygiene Institute in Würzburg; and R. O. Neumann, Dr. Phil. and Med., Assistant in the Hygienic Institute in Würzburg. From the second enlarged and revised German edition. Edited by George H. Weaver, M. D., Assistant Professor of Pathology, Rush Medical College, Chicago. In two volumes. Part I, consisting of 632 colored figures on 69 lithographic plates. Part II, consisting of 511 pages of text, illustrated. Philadelphia and London: W. B. Saunders & Company. 1901. Cloth, $5.00 net.

The text of this work, written chiefly by Dr. Lehman himself, is, we feel safe in assuming, the best and most thoroughly original presentation of the subject which has yet appeared in any language. It covers 511 pages and is divided into I, a general part consisting of a condensed study of the properties of bacteria viewed from a practical standpoint, and II, a special part giving their botanical arrangement, and containing a description of their various varieties.

The splendidly executed colored figures and lithographic plates, of which there are altogether 702, are far superior, for obvious reasons, to the usual photographic representations.

As a text-book, the work is all that could be desired. It will certainly do much to simplify bacteriologic diagnosis. R.

